# Teaching Hands-Only CPR in Schools: A Program Evaluation in San José, Costa Rica

**DOI:** 10.29024/aogh.2367

**Published:** 2018-11-05

**Authors:** Kristin M. Schmid, Raquel Quiros García, Manrique Montoya Fernandez, Nee-Kofi Mould-Millman, Steven R. Lowenstein

**Affiliations:** 1 St. Louis University, Department of Emergency Medicine, St. Louis, MO, US; 2 Universidad de Santa Paula, San José, Costa Rica, US; 3 Universidad Estatal a Distancia, San José, Costa Rica, US; 4 University of Colorado, Department of Emergency Medicine, Aurora, CO, US; 5 Colorado School of Public Health, University of Colorado, Aurora, CO, US

## Abstract

**Background:**

Hands-only bystander CPR increases survival from out-of-hospital cardiac arrest. Video-based CPR instruction in schools has been proposed as a means to mass-educate laypersons in Hands-only CPR™ (HOCPR), in developed as well as developing countries.

**Objectives:**

The purpose of this study is to determine whether a brief video- and mannequin-based instructional program, developed by the American Heart Association (AHA), is an effective strategy for teaching Costa Rican middle- and high-school age children to learn the steps of HOCPR.

**Methods:**

This study took place in four educational centers that spanned the entire socioeconomic spectrum within the Grand Metropolitan Area of Costa Rica. Three hundred and eight students from the sixth to eleventh grades participated. The intervention included exposure to the AHA “CPR Anytime” video and practice with CPR mannequins. Before and after the intervention, students took a four-question, multiple-choice quiz that measured their knowledge of the correct steps and proper techniques of HOCPR; a separate question assessed their level of comfort “doing CPR on someone with a cardiac arrest.” Pre- and post-intervention “percent correct” scores were compared and tested for statistical significance using paired t-tests or the McNemar test as appropriate. Improvement in knowledge and comfort levels were also compared across the different educational centers and compared with similar programs implemented in the United States.

**Results:**

The students’ overall scores (mean percent correct) on the multiple choice questions more than doubled after training (40.9% ± 1.4% before training vs. 92.5% ± 0.9% after training, p < 0.00001). Improvements were observed in each school, regardless of geographic location or socioeconomic status. Knowledge of the appropriate steps of HOCPR doubled after training (42.2% before training vs. 92.5% after training, p < 0.000001). Post-intervention, a majority (73%) of children reported comfort with performing CPR on an individual who had suffered a cardiac arrest.

**Conclusion:**

This study demonstrates the effectiveness of the AHA “CPR Anytime” program in teaching HOCPR to school-age children within the Grand Metropolitan Area of Costa Rica. Additional studies are needed to measure longer-term knowledge retention and students’ ability to perform CPR in simulated cardiac arrest settings.

## Background

Cardiac arrest, defined as the sudden cessation of cardiac activity with hemodynamic collapse, is the principal cause of death for patients with coronary artery disease (CAD) [1]. Survival after cardiac arrest is dependent upon recognition of the arrest, activation of the emergency response system, prompt initiation of bystander-performed chest compressions, early defibrillation and access to advanced life support and post-cardiac-arrest care (the “chain of survival”) [1,2,3,4]. The incidence of bystander cardiopulmonary resuscitation (CPR) depends on recognition of the event, training level, and the willingness of a layperson responder to participate when a cardiac arrest occurs [3,4]. “Willingness” to perform CPR reflects not only training, but also personal experiences, level of education, local cultures and norms, and attitudes, fears and beliefs regarding the risks and benefits of performing CPR on a stranger [5,6,7].

Costa Rica is a middle-income country in Central America, where the leading cause of death is CAD (16.4% in 2015) [8]. The Grand Metropolitan Area (GMA) of Costa Rica, a high-population density area surrounding the capital, San José, includes 50 percent of the Costa Rican population. The GMA has a robust Emergency Medical Services (EMS) prehospital and hospital system capable of managing cardiac arrest patients with Advanced Life Support; however, bystander CPR is rarely performed [9]. In a 2014 study of laypersons in San José, we found a high level of interest in taking CPR classes (55%) and a willingness to perform CPR in the event of a cardiac arrest (74%) [7]. A separate study of San José emergency physicians demonstrated that the vast majority (97%) believe that layperson CPR training would improve morbidity and mortality from out of hospital cardiac arrest (OHCA) under the current San José EMS system [9].

Our earlier study of laypersons in San José, Costa Rica found that while interest in taking CPR classes and willingness to perform CPR are high, they are not universal. Barriers exist, such as a lack of confidence in the effectiveness of CPR; there are also practical barriers to taking CPR classes, such as affordability, accessibility and lack of personnel trained to give CPR classes. Other significant barriers to registering for CPR classes or expressing willingness to perform CPR on a stranger include the fear of legal consequences and hesitancy to perform mouth-to-mouth ventilations [9]. Many of these barriers have also been identified in low-income and Latino neighborhoods in the United States [5,6].

One approach to circumventing the fear of mouth-to-mouth ventilation and decreasing the complexity of bystander CPR has been the implementation of hands-only (compressions-only) CPR education for non-medical persons [10,11]. Hands-only CPR™ (HOCPR) has even been shown to be superior to conventional CPR in certain settings; for example, studies by Cabrini et al. and Hüpfl et al. have shown higher rates of survival among out-of-hospital cardiac arrest (OHCA) victims who receive HOCPR versus conventional CPR [12,13]. In one nationwide study in Japan, over an eight-year period (2005–2012), dissemination of HOCPR training for lay-rescuers was associated with a 47-fold (0.6 to 28.3 per 10 million) increase in survival with favorable neurological outcomes after OHCA [14].

Most conventional and HOCPR training programs have focused on adult learners. However, some countries (for example, Sweden and Norway) have incorporated CPR training as part of the curriculum in schools [15]. The American Heart Association (AHA) and others have suggested that students attending school may be an appropriate audience for CPR training [10,15]. According to the AHA, “teaching CPR to school-aged children at the middle school level and higher is effective and may result in retention of the skills and knowledge” as well as changing the stigma and fear associated with performing CPR [10]. Incorporating CPR instruction into school curricula may be a sustainable way to teach CPR as well as a way to create a critical mass of laypersons within the community capable of performing CPR [10]. One successful study, performed in 1,131 public schools around the United States, utilized the AHA’s “CPR in Schools” kit as a means to educate middle school students, taking advantage of the personnel and infrastructure already in place in the public school system. Seventy-six percent of students stated they were comfortable with performing CPR post-instruction and performed an average of 34% better on a post-instruction quiz assessing knowledge of the correct steps of HOCPR, depth and rate of compressions, when to stop performing compressions, and the function of an Automated External Defibrillator [16].

The current study tests whether a Spanish-language, HOCPR video, developed by the AHA and validated among middle-school children in the United States, is an effective strategy for teaching Costa Rican age- and education-equivalent children to learn the steps of HOCPR.

## Methods

### Setting

This study took place in four educational centers within the Grand Metropolitan Area of Costa Rica in January and February of 2017: Unidad Pedagógica José Fidel Tristán in Merced, San José; Isaac Phillipe in Santo Domingo, Heredia; Casa de Niños in Tirrases, San José; and Casa Hogar San Lázaro in Río Azul, Cartágo. These educational centers were chosen to represent a broad spectrum of socioeconomic diversity. Casa de Niños in Tirrases and the Casa Hogar San Lázaro in Río Azul are two cafeterias that provide meals and workshops for children enrolled in school within their corresponding communities. Río Azul is a *precario* (shanty-town or slum), and Tirrases is a low-income area with populations of 13,500 and 20,100 respectively [17,18]. Unidad Pedagógica José Fidel Tristán is a public school located in Merced District, San José; Merced has a population of 14,700 [19]. The Costa Rican Institute of Census and Statistics uses the “Unsatisfied Basic Needs” method formulated by the Economic Commission for Latin America and the Caribbean (ECLAC) of the United Nations to categorize poverty based on four categories (access to housing, access to education, access to sanitation services and economic capacity) to describe the rates of poverty in each district [20]. Accordingly, Merced District has a 27.7% poverty rate, Tirrases 37.2% and Rio Azul 42.0% [21]. Isaac Phillipe Primary and High School is a private school located in Santo Domingo, Heredia.

### Cohort

The study cohort included sixth through eleventh grade students between the ages of 11 and 18. Only students whose parents had signed a consent form participated in the study; minors with medical conditions limiting strenuous physical activity were discouraged from participating via parental discretion as explained on the consent forms. Each school administrator signed a letter granting permission for the use of his or her school as a project site. Approval was also obtained from the Colorado Multiple Institutional Review Board (Denver, Colorado USA) as protocol #16-1817.

### Intervention and Outcome Measures

HOCPR training was conducted during January and February 2017. Training was conducted by three of the authors (KS, MM and RQ), assisted by members of the school staff. Each school chose one to two days during which students would participate in the study. The intervention consisted of exposure to the AHA “CPR Anytime” video (identical to the “CPR in Schools” video used in the United States public schools study), and a 13-minute review of each of the steps of responding to a cardiac arrest, including identifying a cardiac arrest, calling 9-1-1, and initiating compressions at a rate of 100/min and depth of 5 cm. After watching the video and asking questions of the instructors, students were given approximately 15 minutes to practice HOCPR skills on inflatable, instant-feedback CPR mannequins. Instructors were present during the practice session to provide additional guidance with regard to compression technique (hand position and depth and rate of compressions), in order to solidify the conceptual content and maximize confidence performing HOCPR.

Knowledge acquisition was assessed by measuring participants’ responses to a four-question multiple-choice quiz. The quiz, administered before and after the video and mannequin intervention, was a Spanish translation of the instrument used in the United States school-based CPR training study [16]. The quiz was written at a sixth-grade level and was translated into Spanish and validated for use in Costa Rican schools after local expert input. The knowledge questions addressed the rate of compressions, depth of compressions, correct steps of HOCPR and when CPR should be discontinued. An additional question measured students’ “level of comfort” in performing CPR on someone in cardiac arrest. A single question about Automatic External Defibrillators (AEDs) from the Denver study was excluded, due to lack of widespread AED availability within the Grand Metropolitan Area.

The primary outcome measure was the change in the percentage of questions answered correctly after participation in the video and mannequin practice sessions. Secondary outcomes included: change in level of comfort in performing HOCPR after the intervention; knowledge of the correct steps of HOCPR after the intervention; and differences between the percent improvement of United States and Costa Rican students of the same age.

### Data Analysis

Each participant served as his or her own control. Students were assigned unique identification numbers, so that test performance before and after the intervention could be compared. Performance on the test was measured by calculating the “percent correct” for the four knowledge-based questions. The “level of comfort” question was analyzed separately. To measure the overall impact of the video- and practice-based instruction, pre-intervention and post-intervention “percent correct” were compared and tested for statistical significance using paired t-tests. Only test scores from students who completed tests before and after the intervention were included in the analysis. Questions left blank or with multiple answers chosen were marked as “incorrect.” Pre- and post-intervention data (“percent correct”) were analyzed for all four knowledge questions combined, and also separately for each question, in order to identify areas of learning that were more or less responsive to the classroom instruction. For individual questions, pre-intervention and post-intervention results were compared using the McNemar test. Also, data were analyzed separately for each of the four participating centers.

## Results

Three hundred and eight students, all ages 11–18 and enrolled in grades six through eleven, participated in the HOCPR classes. Fifty-one students were from Rio Azul, 13 from Tirrases, 93 from Isaac Phillipe, and 151 from José Fidel Tristán. All students completed quizzes before and after the intervention and were included in the analysis.

When considering all 308 students and the four questions combined, the students’ scores (mean percent correct) more than doubled after training (40.9% ± 1.4% before training vs. 92.5% ± 0.9% after training, p < 0.00001). Similar, statistically significant improvements were observed in each school, regardless of geographic location or poverty rate: Río Azul (46.1% ± 3.8% vs. 87.3% ± 2.8%, p < 0.00001); Tirrases (32.7% ± 6.6% vs. 78.9% ± 7.9%, p = 0.00014); Isaac Phillipe (37.6% ± 2.9% vs. 95.4% ± 1.0%, p < 0.00001); and José Fidel Tristán (42.1% ± 1.9% vs. 93.7% ± 1.1%, p < 0.00001) (Table 1).

**Table 1:** Improvement in Overall CPR Test Scores After Video and Manikin Training.

School (# of students)	Pre-test Average ± SEM (%)	Post-test Average ± SEM (%)	p-value	Difference in mean scores (%)	95 CI

Río Azul (51)	46.1 ± 3.8	87.3 ± 2.8	p < 0.00001	41.2	32.88, 49.48
Tirrases (13)	32.7 ± 6.6	78.9 ± 7.9	p = 0.00014	46.2	27.81, 64.50
José Fidel Tristán (151)	42.1 ± 1.9	93.7 ± 1.1	p < 0.00001	51.6	47.56, 55.75
Isaac Philippe (93)	37.6 ± 2.9	95.4 ± 1.0	p < 0.00001	57.8	51.92, 63.67
Overall (308)	40.9 ± 1.4	92.5 ± 0.9	p < 0.00001	51.5	48.43, 54.65

Table 2 shows that students demonstrated improvement across all questions. Importantly, knowledge of the appropriate steps of HOCPR more than doubled after training (42.2% before training vs. 92.5% after training; p < 0.000001). After training, almost three-fourths (73%) of children reported they “would be comfortable” performing CPR on an individual who had suffered a cardiac arrest (Table 3).

**Table 2:** Percent Correct Pre- And Post-Video, Facilitator Review And Manikin Training: Specific Knowledge Questions.

Quiz Question	% Correct Before (Number)	% Correct After (Number)	P value

1. Number of compressions per minute	15.5(48)	94.2(290)	<0.000001
2. When to stop compressions	50.0(154)	89.3(275)	<0.000001
3. Depth of compressions	24.0(74)	93.5(288)	<0.000001
4. Correct order of steps	42.2(230)	92.5 (285)	<0.000001

**Table 3:** Change in Comfort Level Pre- and Post-Video and Manikin Training.

Change in level of comfort	% of Students (Number)

No➔No	21.4% (66)
No➔Yes	20.5% (63)
Yes➔Yes	52.6% (162)
Yes➔No	6.5% (17)

## Discussion

This study provides new information about the effectiveness of the American Heart Association’s “CPR Anytime” training program in improving CPR knowledge and confidence among middle and high school students in a middle-income Central American country. A brief intervention, consisting of an AHA video and hands-on manikin practice, significantly improved students’ knowledge of the steps involved in performing HOCPR.

Coronary Artery Disease (CAD) has become a major cause of morbidity and mortality among low- to middle-income countries in recent years, and in Costa Rica it has replaced trauma and infectious diseases as the principal cause of death [8]. In high-income countries, the morbidity and mortality from cardiac arrest, most commonly the result of CAD, has been reduced by training laypersons to perform CPR [1,14,15,22,23]. Of course, layperson (bystander)-performed CPR is only one of the critical links in the chain of survival. There must also be an EMS system that can respond to cardiac arrest patients with Advanced Life Support and trained personnel, and definitive treatment options must be available within the hospitals [4,23].

The Grand Metropolitan Area of Costa Rica is one of the places that could benefit from mass layperson CPR training, given its well-developed pre- and in-hospital emergency care system capable of providing advanced life support and post-arrest care. Currently in the public ambulance sector, there are 24 bases of the Cruz Roja (Red Cross); two have 24 hours-per-day advanced life support, and five have advanced life support during certain hours. There are also 19 stations of bomberos (firefighters); four have 24-hours-per-day ALS ambulances.

Before implementation of a community CPR education program, cultural acceptability and local expert input must also be considered. Our group previously carried out a series of layperson and emergency physician focus groups and surveys to learn more about the barriers and facilitators to community CPR education in San Jose [7,9]. We found that a majority of adult laypersons would be willing to take a CPR class (55%) and perform CPR on a cardiac arrest victim (73%), with few perceived barriers [7]. Importantly, a majority of local emergency physicians believed that community CPR education would be reduce morbidity and mortality from cardiac arrest within the current EMS system [9].

Given this local context, our group designed a pilot program based on a similar project carried out among middle-school students in Denver and Aurora, Colorado. The rationale is that schools may offer an efficient mechanism to reach large numbers of individuals to create a “critical mass” of laypersons trained in, and comfortable performing, CPR. School-based CPR training takes advantage of the existing educational personnel and facilities [10]. Importantly, the AHA “CPR Anytime” kits are designed to help non-medical personnel learn HOCPR, without the presence of a trained or certified instructor. Thus, these tools represent a resource-friendly way to disseminate important information to large numbers of people, requiring only an initial monetary investment. The “CPR Anytime” course does not seek to provide “certification;” rather, it teaches a simplified version of HOCPR, focusing on recognizing a cardiac arrest, calling the emergency response number and initiating compressions until arrival of an ambulance.

One strength of the current study is that the four schools represent a broad range of socioeconomic conditions. Rio Azul and Tirrases represent some of the poorest neighborhoods in the GMA, while the children who attend Isaac Phillipe tend to come from upper class families. Our study found that the educational intervention succeeded in all sites. We saw improvement in test scores (a significant doubling) and a high level of comfort (73%) performing HOCPR post-intervention in all groups. We sought to test the intervention in diverse communities because, traditionally, CPR education has been limited to individuals with the resources to pay for a class and the ability to take time from work to attend one [5,6,10,11]. This study provides support for the cognitive effectiveness of HOCPR training, even in poorer communities where few individuals are able to attend or pay for formal classes. Our study also showed that the improvement in pre- and post-tests and levels of comfort post-intervention were similar for both Costa Rican and United States students. The United States pre- and post-test averages were 50% and 84%, respectively, as compared with the Costa Rican pre- and post-test averages of 41% and 93%. The levels of comfort after the classes for United States and Costa Rican students were 76% and 73%, respectively [16].

This study also has several limitations. First, it was conducted in a single metropolitan area in one country in Central America; the results may be different in other communities. Second, we only measured outcomes immediately after the intervention; the improvements in knowledge that we observed may not be sustained. The measured outcomes were also limited to an assessment of graded, written quizzes; psychomotor skills were not tested. A third limitation was the presence of researchers with prior CPR training during the CPR classes. Finally, even before the video and mannequin intervention, a relative high proportion of students (59.1%) reported they felt comfortable performing CPR on a cardiac arrest victim.

Although this study demonstrated the effectiveness of the “CPR Anytime” video and mannequin intervention among middle and high school children in the Grand Metropolitan Area of Costa Rica, future studies should test the effectiveness of train-the-trainer programs, in which local health educators are taught how to teach the classes in their respective schools. Additionally, a school-based CPR educational program may not be sustainable in these Costa Rican communities without buy-in and support from the bomberos, the Ministerio de Educación, the Cruz Roja or other community organizations.

Several obstacles and ethical challenges need to be considered before implementation of HOCPR education programs in low-income or resource-poor communities. Video-based educational tools require televisions, computers and other projection equipment that may be difficult to find in some areas. There must be a reliable source of electricity; during one of our team’s teaching days in Rio Azul, an electrical outage forced a postponement of the classes. In addition, the Grand Metropolitan Area is a unique site in Central America, in that it has a well-developed pre-hospital system capable of providing advanced life support to OHCA victims within a reasonable time frame [23]. In areas that do not have access to ambulances or trained personnel within a short window of time, the probability of survival from OHCA with good neurological outcome is vastly decreased. In these communities it may be unethical to expend resources to teach HOCPR to laypersons. Another consideration in many resource-poor communities is the relative unavailability of Automatic External Defibrillators, which are a proven adjunct to improve survival after OHCA.

## Summary

Short, video- and mannequin-based CPR instruction may be a viable way to mass educate school-age children in low-resource areas where CAD represents a significant burden of disease and where there are pre-hospital and hospital systems capable of managing OHCA victims. Additional studies are needed to measure students’ CPR skills, longer-term knowledge retention and their ability to perform CPR in simulated cardiac arrest settings.

## Additional File

The Additional File for this article can be found as follows:

10.29024/aogh.2367.s1 Quiz. Pre- and post-intervention quiz provided to study participants.
