# Supplementary material for: Teaching Hands-Only CPR in Schools: A Program Evaluation in San José, Costa Rica
Source: Ann Glob Health. 2018 Nov 5;84(4):612–7. doi: 10.29024/aogh.2367 (PMC6748223; doi:10.29024/aogh.2367)
Supplement: Quiz. — Pre- and post-intervention quiz provided to study participants. [file agh-84-4-2367-s1.pdf]

Quiz*

1. When you are doing CPR, how many times per minute should you push on the center of a person's chest?
    a. At least 50
    b. At least 150
    c. At least 100
    d. I don't know

2. When should you stop pushing on a person's chest during CPR?
    a. 5 minutes
    b. Until I get tired
    c. Until the health professionals arrive
    d. I don't know

3. How deep should you push on the chest of an adult during CPR?
    a. 2.5 cm
    b. 5 cm
    c. 7.5 cm
    d. I don't know

4. What are the correct steps of "hands-only" CPR?
    a. Call 9-1-1 and then push hard and fast on the center of the person's chest until a health professional arrives
    b. Push hard and fast on the center of the chest and then call 9-1-1
    c. Give the person 2 breaths mouth-to-mouth and then call 9-1-1
    d. I don't know

5. Would you feel comfortable doing CPR on someone who had a cardiac arrest?
    a. Yes
    b. No

*Correct answers for 1, 2, 3, and 4 are (c), (c), (b), and (a), respectively
